# The relationship between fear of surgery and affecting factors in surgical patients

**DOI:** 10.1186/s13741-023-00316-0

**Published:** 2023-06-09

**Authors:** Seda Akutay, Özlem Ceyhan

**Affiliations:** 1 Department of Surgical Diseases Nursing, Erciyes University Faculty of Health Sciences, Kayseri, 38030 Turkey; 2 Department of Internal Medicine Nursing, Erciyes University Faculty of Health Sciences, Kayseri, 38030 Turkey

**Keywords:** Pre-operative period, Fear of surgery, Surgery, Nursing

## Abstract

**Background:**

This study aimed to explain the fear of surgery in surgical patients, the affecting factors, and their relationship.

**Methods:**

This study was conducted as a descriptive and cross-sectional study. The study population consists of 300 patients undergoing surgical intervention. Data were collected using the “patient information form” and “Surgical Fear Questionnaire.” Parametric and nonparametric tests were used to evaluate the data. The relationship between the fear questionnaire and age, number of previous surgeries, and pre-operative pain was evaluated using Spearman correlation analysis. The relationship with emotional stress was evaluated with multiple linear regression analysis.

**Results:**

In this study, it was determined that the predictors of the surgical fear level of the patients were age, gender, anesthesia type, and pre-operative pain experience. There was a negative correlation between the age of the patients and the fear of surgery score and a positive correlation between the pre-operative pain severity and the fear of surgery score. It was determined that the factors most associated with pre-operative fear levels were the patients’ pre-operative sense of inadequacy (*p* < 0.001), feeling anxious and unhappy, and confusion about the surgery decision (*p* < 0.05).

**Conclusion:**

According to the results of this study, it has been determined that the emotional states and fears of the patients before the surgery have significant effects on the fear of surgery. For this, it is recommended to determine the emotional states and fears of the patients before the surgery and to make appropriate interventions, as it will facilitate compliance with the surgical process.

**Supplementary Information:**

The online version contains supplementary material available at 10.1186/s13741-023-00316-0.

## Background

Having surgery is an experience that creates anxiety and fear in individuals. Pre-operative fear is a typical emotional response in many patients awaiting surgery (Theunissen et al. 2018). Anxiety can be seen in 60–80% of patients before surgery (Guerrier et al. 2021; Karayağız et al. 2011). The reasons for the anxiety of the patients before the surgery are uncertainty about the disease and the future, lack of information about the operation process, inability to wake up from anesthesia, lifestyle changes, deterioration in body image, postoperative pain, disability, and fear of death (Karadağ et al. 2019).

Anxiety and fear before surgery can affect postoperative wound healing, pain and anesthesia intensity, and analgesia requirements (Stamenkovic et al. 2018; Maeshi et al. 2018; Sidar et al. 2013). In this process, the stress response affects wound healing directly and indirectly. The direct effect is due to stress hormones (cortisol, epinephrine, norepinephrine), while the indirect effect is due to the type of anesthesia, general health status before the surgery, and the effects of habits such as smoking and alcohol (Karadağ et al. 2019; Sürme 2019; Caumo et al. 2001).

Emotional well-being in the pre-operative period also affects physiological and psychological recovery in the postoperative period. In the literature, the level of anxiety and fear in surgical patients was found to be higher in women, those who came to surgery alone, patients who would undergo major surgery such as cardiac and vertebral surgery, young people, and those with bad anesthesia experience (Stamenkovic et al. 2018; Cimilli 2001; Fındık and Yıldızeli 2012; Karadağ 2017; Arslan et al. 2017; Kaynar şimşek A, Şimşek T, Ecevit Alpar Ş. 2018; Çelik and Edipoğlu 2018). For this reason, planning the surgical process by considering these factors in the pre-operative period is essential.

Many studies deal with patients’ emotional, social, and individual factors and their effects on various health parameters. It is also argued that these factors may affect the course of acute and chronic disease and the recovery and survival of patients (Levett 2019; Mavros et al. 2011). In addition, it is stated in the literature that pre-operative anxiety and fear increase the intensity of postoperative pain and cause the individual to need more analgesics and have difficulty controlling pain (Sidar et al. 2013; Robleda et al. 2014; Socea et al. 2020; Peker 2020; Taşdemir et al. 2013; Kandemir et al. 2019).

It is imperative to reveal the factors affecting the fear and explain the relationships of these factors in planning the right interventions for the patients to make the proper intervention regarding the patients’ fears in the pre-operative period. However, studies examining the relationship between these factors, which are thought to affect pre-operative fear, are limited in the literature (Sürme and Cimen 2022; Taylan and Çelik 2022; Amiri et al. 2021; Kaya and Karaman 2019). In addition, there is no study in which emotional reactions to surgical intervention are revealed and the relationship between fear levels. This study explained the pre-operative fear levels of patients undergoing surgical intervention and the relationship between the affecting factors.

## Methods

This was a cross-sectional, descriptive, and correlational study. The study was conducted between February and May 2021 with inpatients in the surgical clinics (brain and neurosurgery, general surgery, thoracic surgery, orthopedics and traumatology, urology) of a hospital with a 679-bed capacity and the pre-operative period for surgical procedures. Using G*Power 3.1.9.4 software to calculate the sample size, the number of people to be sampled was determined as 262 with 95% power and 5% type 1 error rate (Işıklı et al. 2022), but 300 people were included in the sample considering the data losses.

To determine the fear of surgery more objectively, the surgical interventions to be applied to patients with an equal number of minor and major surgeries from each surgery clinic were included (Table 1. The surgical classification was based on previous studies that considered various criteria such as blood loss, degree of pain and opening of a body cavity (e.g., abdomen, chest), need for monitoring, and length of hospital stay (Stavrou et al. 2014; Caumo et al. 2016).

**Table 1:** Surgical classification of patients included in the study

Surgery classification	
**Minor surgery (*n* = 150)**	• In neurosurgery, general surgery, thoracic surgery, orthopedics and traumatology, urology services
	• Pleural surgeries, cholecystectomy, minor head trauma, minor cyst surgery, transurethral resection, hernia surgery, minimally invasive thoracic surgery, thyroidectomy, colostomy closure, breast reconstruction, external fixator removal, percutaneous nephrolithotomy, etc
**Major surgery (*n* = 150)**	• In neurosurgery, general surgery, thoracic surgery, orthopedics and traumatology, urology services
	• Oncological surgeries, Whipple, total hip replacement, total knee replacement, fracture surgeries, prostatectomy, spinal surgery, mastectomy, cystectomy, pneumonectomy, spine surgery, gastrectomy, pancreatectomy, hepatectomy, etc

Inclusion criteria for the study is as follows: (1) 18 years of age or older, (2) elective surgery is planned and is in the pre-operative process, (3) ASA I-III classified, and (4) can communicate in Turkish. Patients with a history of using drugs (antidepressant, antipsychotic, anxiolytic) affecting the central nervous system, a psychiatric disease, mental retardation, and did not want to participate in the study were excluded from the study.

**Fig. 1:**
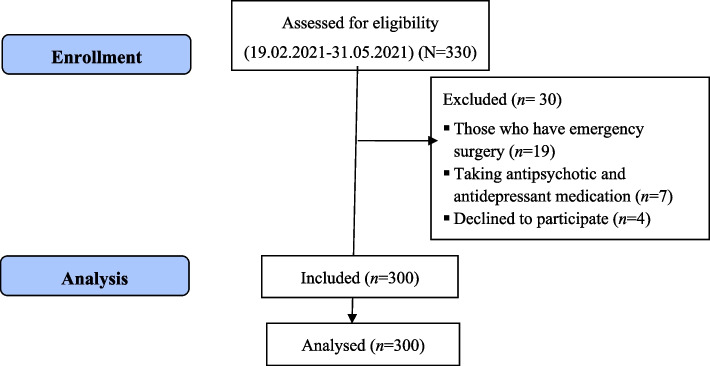
CONSORT flow diagram

Between the data collection dates, 330 patients were included in the study. However, the study was terminated with 300 patients because seven had used antipsychotic and antidepressant drugs, four refused to participate, and 19 had undergone emergency surgery (Fig. 1).

### Data collection and tools

In this study, data were collected using the patient information form and the Surgical Fear Questionnaire.

#### Patient information form

The researchers create questions to determine the patients’ demographic data, the data about the surgery, and the emotional stress. Stress-related questions were formed by using the “The Brief Measure of Emotional Preoperative Stress Scale” (Caumo et al. 2016). Pre-operative stress questions were asked in a yes/no format.

#### Surgical Fear Questionnaire (SFQ)

This determines the level of fear of patients who will undergo elective surgery (Theunissen et al. 2014). A Turkish validity and reliability study was conducted in 2018 (Bağdigen and Karaman 2018). The questionnaire, consisting of eight items, has an 11-Likert structure. Each item is scored as 0, “not afraid at all,” and 10, “very afraid.” The questionnaire has two subdimensions, each consisting of four items, showing the fear of surgery’s short- and long-term results. The subscale score is obtained by adding the scores of the four items in the subdimensions of the questionnaire, and the total score of the questionnaire is formed by adding the scores of the two subscales. The total questionnaire score is 0 at the lowest and 80 at the highest. A high score indicates a heightened fear of surgery. In this study, the mean score of the Surgical Fear Questionnaire was 18.04 ± 20.38, the mean score of the short-term subdimension (SFQ-S) was 9.49 ± 10.86, and the mean score of the long-term subdimension (SFQ-L) was 8.55. ± 11.58. The Cronbach’s a coefficient of the adapted scale was 0.93 (Bağdigen and Karaman 2018). In our study, the Cronbach a coefficient of the scale was 0.88.

In data collection, patients were visited in the patient rooms of the clinic the day before the surgery, the purpose of the study was explained, and informed consent was obtained from the individuals who agreed to participate in the study. In the pre-operative period, the patient information form and SFQ were applied to the patients. It took an average of 5–7 min for the patients to respond to the questionnaires.

### Statistical analysis

Data analysis was performed using the SPSS (Statistical Package for the Social Sciences) 24.0 program. The conformity of the data to the normal distribution was checked with kurtosis-skewness. To compare descriptive statistics and groups, independent samples *t*-test and ANOVA were used for normally distributed data, and Mann–Whitney *U* and Kruskal–Wallis tests were used for data that did not conform to normal distribution. The relationship between scale total score and sub-dimension scores, age, number of previous surgeries, and pre-operative pain was evaluated by Spearman correlation analysis. The effect of items measuring the emotional stress of the patients on the fear scale total score was evaluated with multiple linear regression analysis. In all results, *p* < 0.05 was considered statistically significant.

## Results

The mean age of the patients in this study was 54.29 ± 14.45 (18–88). It was determined that 56% of the patients were male, 76.7% had previous surgery, and 31.3% of those who had surgery had more than three surgeries. A total of 50.7% of the patients were hospitalized for major surgery, open surgery was planned for 44.7%, general anesthesia was applied to 89.0%, 39.7% experienced pain, and 23.53% of the patients with pre-operative pain felt pain of 7–10 severity (Table 2).

**Table 2:** Basic characteristics of the patients

Characteristics	Mean ± SD
**Age (year) (min–max)**	54.29 ± 14.45 (18–88)
**Age groups**	***n* (*%)***
18–44 years old	71 (23.7)
45–64 years old	145 (48.3)
65 years and older	84 (28.0)
**Gender**	
Female	132 (44.0)
Male	168 (56.0)
**History of surgery**	
Yes	230 (76.7)
No	70 (23.3)
**Number of previous surgeries (*n* = 230)**	
One	99 (43.04)
Two	59 (25.65)
Tree or more	72 (31.30)
**Classification of current surgery**	
Minor surgery	148 (49.3)
Major surgery	152 (50.7)
**Type of current surgery**	
Open surgery	134 (44.7)
Laparoscopic surgery	166 (55.3)
**Type of current anesthesia**	
General anesthesia	267 (89.0)
Epidural anesthesia	33 (11.0)
**Pre-operative pain**	
Yes	119 (39.7)
No	181 (60.3)
**Pre-operative pain severity (*n* = 119)**	
Between 1 and 3	44 (36.97)
Between 4 and 6	47 (39.49)
Between 7 and 10	28 (23.53)

**Fig. 2:**
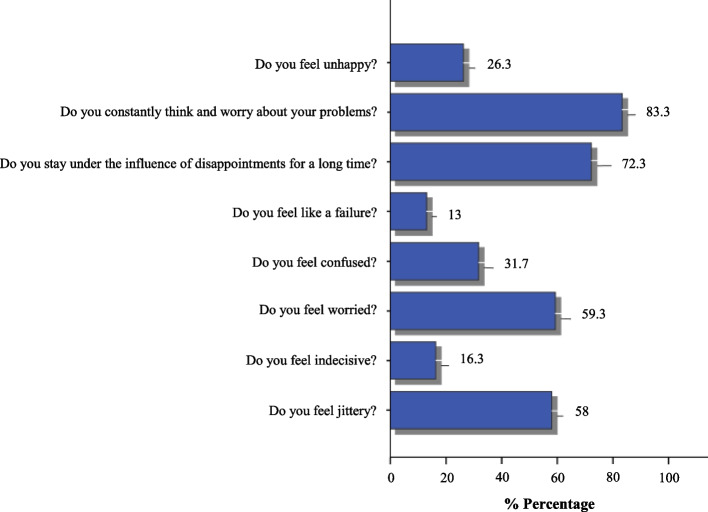
Questions about the emotional stress of the patients (*n* = 300)

According to the answers to the questions asked to determine the emotional stress levels before the surgery, 58% of the patients felt jittery, 16.3% were indecisive about having the surgery, 59.3% were worried, and 31.7% were confused. It was determined that 13% felt failure about surgery, 72.3% remained under the influence of disappointments for a long time, 83.3% constantly thought about their problems and worried, and 26.3% felt unhappy before the surgery (Fig. 2).

When the relationship between the basic characteristics of the patients participating in the study and the surgical fear questionnaire and sub-dimension average score was examined, it was determined that the fear ranking average score of the patients aged 18–44 years, female, who had surgery in neurosurgery and thoracic surgery clinics was higher. The fear scale mean score of the patients who were to be operated on under general anesthesia and who had severe pain before the operation is higher (*p* < 0.05) (Table 3).

**Table 3:** The relationship between the descriptive and surgical characteristics of the patients and the fear of surgery scale

	**SFQ-S**	**SFQ-L**	**SFQ total**
**Age groups**	**x̄ ± SD**	**x̄ ± SD**	**x̄ ± SD**
18–44 years old	12.41 ± 11.49	11.48 ± 12.35	23.89 ± 22.19
45–64 years old	9.15 ± 10.24	7.62 ± 10.98	16.77 ± 18.93
65 years and older	7.61 ± 10.98	7.68 ± 11.67	15.29 ± 20.51
p*	**0.020**	**0.051**	**0.018**
**Gender**			
Female	13.69 ± 11.83	10.92 ± 12.55	24.62 ± 21.99
Male	6.18 ± 8.74	6.68 ± 10.44	12.86 ± 17.43
p**	**0.000**	**0.002**	**0.000**
**Surgical clinic**			
Orthopedics	9.50 ± 11.06	8.63 ± 11.38	18.13 ± 20.18
General surgery	9.00 ± 10.20	6.68 ± 10.23	15.68 ± 18.88
Brain surgery	11.11 ± 11.27	10.85 ± 12.18	21.95 ± 21.71
Urology	7.22 ± 10.37	6.60 ± 10.88	13.83 ± 19.69
Thoracic surgery	11.33 ± 11.98	11.63 ± 14.43	22.96 ± 23.28
p***	0.156	**.045**	**.040**
**History of surgery**			
Yes	9.53 ± 10.86	8.68 ± 11.54	18.21 ± 20.19
No	9.34 ± 10.94	8.13 ± 11.82	17.47 ± 21.15
p**	0.897	0.729	0.790
**Type of current surgery**			
Open surgery	10.09 ± 10.73	8.647 ± 11.45	18.76 ± 20.38
Laparoscopic	9.00 ± 10.98	8.45 ± 11.73	17.45 ± 20.43
p**	0.389	0.867	0.579
**Classification of current surgery**			
Minor surgery	10.54 ± 11.32	8.96 ± 11.87	19.50 ± 20.98
Major surgery	8.47 ± 10.33	8.15 ± 11.33	16.62 ± 19.76
p****	0.089	0.628	0.172
**Type of current anesthesia**			
General anesthesia	10.08 ± 11.05	9.19 ± 11.93	19.27 ± 20.88
Epidural anesthesia	4.73 ± 7.85	3.36 ± 6.34	8.09 ± 12.10
p****	**0.005**	**0.007**	**0.001**
**Pre-operative pain**			
Yes	12.13 ± 12.12	10.47 ± 12.85	22.60 ± 22.98
No	7.76 ± 9.59	7.29 ± 10.52	15.04 ± 17.92
p**	**0.001**	**0.025**	**0.003**
**Pre-operative pain intensity**			
Between 1 and 3	8.39 ± 9.96	7.49 ± 10.55	15.88 ± 18.30
Between 4 and 6	10.89 ± 12.23	9.72 ± 12.51	20.62 ± 23.46
Between 7 and 10	15.96 ± 13.08	15.11 ± 15.53	31.07 ± 25.68
p***	**0.006**	0.068	**0.011**

^*^One-way ANOVA. **Independent sample *t*-test. ***Kruskal–Wallis test. ****Mann–Whitney *U*-test

In the Spearman correlation analysis, in which the relationship between the total fear scale score of the patients participating in the study and age, the number of previous surgeries, and pre-operative pain severity were examined, a negative correlation was found between age and fear score and a weak-positive correlation between pre-operative pain severity and fear score (*p* < 0.05) (Table 4).

**Table 4:** The relationship between the Surgical Fear Questionnaire and some variables

	**x̄ ± SD (min–max)**	***p***	***r***^**s**^
Age (year)	54.29 ± 14.45 (18–88)	**0.010**	− 0.148
Number of previous surgeries	1.93 ± 2.76 (0–30)	0.301	0.537
Pre-operative pain intensity	1.93 ± 2.77 (0–10)	**0.004**	0.168

*r*^s^, Spearman correlation analysis

**Table 5:** Multiple linear regression analysis of the Surgical Fear Questionnaire total score and some independent variables

Independent variables	B	SE	*β*	*t*	*p*	95% confidence interval	
						**Lower limit**	**Upper limit**
Do you feel jittery?	5877	5.331	0.143	1.606	0.109	− 1.327	13,081
Do you feel indecisive?	5580	3.660	0.101	1919	.056	− 0.143	11,303
Do you feel worried?	7.688	2.908	0.186	2079	**.038**	0.411	14,965
Do you feel confused?	5150	3.698	0.118	2066	**.040**	0.245	10,056
Do you feel like a failure?	16,442	2492	0.272	5.449	**.000**	10,503	22,380
Do you stay under the influence of disappointments for a long time?	3210	3017	.071	1.151	0.251	− 2280	8700
Do you constantly think and worry about your problems?	2.303	2789	.042	0.704	0.482	− 4135	8741
Do you feel unhappy?	4747	3.271	0.103	2.034	**.043**	0.153	9340

*R*^2^_corrected_ = 0.47. *B* non-standardized regression coefficient, *SE* standard error, *β* standardized regression coefficient. *p* < 0.05. It was determined that the regression model was statistically significant in the multiple linear regression analysis in which the relationship between the patients' pre-operative emotional stress and their pre-operative fear levels was examined (*p* < 0.001; R2 adjusted = 0.47). It was determined that the factors most associated with pre-operative fear levels were the patients' pre-operative sense of failure (*p* < 0.001), feeling worried and unhappy, and confusion about the surgery decision (*p* < 0.05) (Table 5)

## Discussion

Surgery is an experience that causes many patients emotional reactions such as fear and anxiety. The lifetime prevalence of fear or anxiety arising from surgical procedures and/or medical interventions is 12.8% (Schmid et al. 2009; Becker et al. 2007). This study discusses the pre-operative fear levels of the patients undergoing surgical intervention and the relationship between the affecting factors.

In the literature, it is seen that anxiety and fear experienced in the pre-operative period vary depending on age, gender, marital status, education level, type of surgery, anesthesia, fear of postponing the surgery, fear of waking up early from anesthesia, fear of postoperative pain, fear of financial loss, and fear of death (Çelik and Edipoğlu 2018; Sürme and Cimen 2022; Kaya and Karaman 2019; Işıklı et al. 2022; Sigdel 2015; Ghimire and Poudel 2018; Kuzminskaitė et al. 2019; Erkilic et al. 2017; Ruhaiyem et al. 2016). In this study, following the literature, it was determined that young patients had a higher level of fear than older patients, and female patients had a higher level of fear than men. The lower level of fear of elderly patients can be attributed to their familiarity with the hospital environment. After all, they apply to the hospital more frequently due to chronic diseases (Oymaagaclıo and Ates 2019) and the fact that they experience less fear because they see old age as approaching death and accepting it. It is thought that women have higher levels of fear than men may be related to the effects of estrogen-progesterone hormones on emotional changes (Li and Graham 2017; Albert and Newhouse 2019) and to psychosocial stress, such as family responsibilities and inability to fulfill their caregiver role in the family. In addition, although men are reluctant to express their emotions, women’s more comfortable expression of emotions (Masjedi et al. 2017) may have revealed this result. The study determined that the fear level of patients who underwent surgery in clinics such as neurosurgery and thoracic surgery under general anesthesia was higher. It can be said that this situation develops due to the inability to wake up from general anesthesia and the fear of being disabled after surgery. The study is similar to the literature (Alodaibi et al. 2018; Roublah et al. 2020).

Many patients may fear experiencing pain in the postoperative period (Ustunel and Erden 2022). This situation may cause patients to be afraid of having surgery and to postpone the decision or to be indecisive about it. It is seen in the literature that this fear is higher in patients who have severe pain in the pre-operative period (Theunissen et al. 2014; Hoofwijk et al. 2015). In this study, it was seen that 39.7% of the patients had pain, and 23.53% had severe pain before the surgery. It was determined that these patients’ fear questionnaire mean scores were higher than the others. The study is similar to the literature.

The study determined that the factors most associated with pre-operative fear levels were pre-operative anxiety, unhappiness, confusion about the surgery decision, and having a sense of inadequacy. These patients experienced higher fear of surgery. Not having enough information about the pre-operative and postoperative process and being prone to anxiety as a personality can be the reason for these feelings. In addition, it can be expected that the neuroendocrine response caused by anxiety and fear will change physiological parameters, and these feelings will deepen. This may lead to difficulty adapting to the treatment process, an increase in postoperative complications, prolongation of the healing process, and delayed discharge (Bansal and Joon 2016; Şahin Altın et al. 2017; Manou-Stathopoulou et al. 2019; Chen et al. 2022; Kil et al. 2012; Ralph and Norris 2018).

## Conclusions

In this study, it was determined that female patients, young people, patients receiving general anesthesia, and patients with high pre-operative pain had a higher fear of surgery, and pre-operative anxiety, unhappy, confused, and feelings of inadequacy were essential factors associated with fear of surgery. For this, it is recommended to determine the emotional states and fears of the patients before the operation and to make appropriate interventions, as it will facilitate compliance with the surgical process.

## Supplementary Information

**Additional file 1.**

## Data Availability

The datasets used and/or analyzed during the current study are available from the corresponding author on reasonable request.
