# Supplementary material for: The relationship between fear of surgery and affecting factors in surgical patients
Source: Perioper Med (Lond). 2023 Jun 9;12:22. doi: 10.1186/s13741-023-00316-0 (PMC10251663; doi:10.1186/s13741-023-00316-0)
Supplement: Supplementary file 1 — Additional file 1. [file 13741_2023_316_MOESM1_ESM.pdf]

KLİNİK ARAŞTIRMALAR ETİK KURULU KARAR FORMU

ARAŞTIRMANIN AÇIK ADI	Preoperatif Duygusal Stres Ölçeği (Kısa Form): Türkçe Geçerlik ve Güvenirlik Çalışması
VARSA ARAŞTIRMANIN PROTOKOL KODU	

DEĞERLENDİRİLEN BELGELER

BELGE ADI	Tarihi	Versiyon Numarası	Dili		
ARAŞTIRMA PROTOKOLÜ			Türkçe	İngilizce	Diğer
BİLGİLENDİRİLMİŞ GÖNÜLLÜ OLUR FORMU			Türkçe	İngilizce	Diğer
OLGU RAPOR FORMU			Türkçe	İngilizce	Diğer
ARAŞTIRMA BROŞÜRÜ			Türkçe	İngilizce	Diğer

DEĞERLENDİRİLEN DİĞER BELGELER

BELGE ADI		Açıklama
SİGORTA		
ARAŞTIRMA BÜTÇESİ		
BİYOLOJİK MATERYEL TRANSFER FORMU		
İLAN		
YILLIK BİLDİRİM		
SONUÇ RAPORU		
GÜVENLİK BİLDİRİMLERİ		
DİĞER		

ASLI GİBİDİR
Şerife SERİM
Etik Kurul Sekreteri

KARAR BİLGİLERİ

Karar No : 2021/132 Tarih : 17.02.2021

Yukarıda bilgileri verilen prospektif başvuru dosyası ile ilgili belgeler araştırmanın/çalışmanın gerekçe, amaç, yaklaşım ve yöntemleri dikkate alınarak incelenmiş ve uygun bulunmuş olup araştırmanın/çalışmanın başvuru dosyasında belirtilen merkezlerde gerçekleştirilmesinde etik ve bilimsel sakınca bulunmadığına toplantıya katılan etik kurul üye tam sayısının salt çoğunluğu ile karar verilmiştir.

KLİNİK ARAŞTIRMALARI ETİK KURULU

ETİK KURULUN ÇALIŞMA ESASI	Klinik Araştırmalar Hakkında Yönetmelik, İyi Klinik Uygulamalar Kılavuzu
ETİK KURUL BAŞKANI UNVANI/ADI/SOYADI	Prof. Dr. Sema Kader KÖSE

Unvanı / Adı Soyadı	Uzmanlık Alanı	Kurumu	Cinsiyeti E	Cinsiyeti K	Araştırma İle İlişki E	Araştırma İle İlişki H	Katılım (*) E	Katılım (*) H	İmza
Prof. Dr. Sema Kader KÖSE	Tıbbi Biyokimya	E.Ü. Tıp Fak.		x		x	x		
Prof. Dr. Ahmet ÖZTÜRK	Halk Sağlığı	E.Ü. Tıp Fak.	x			x	x		
Doç. Dr. Yusuf SEVİM	Genel Cerrahi	Kayseri Eğitim Hast.	x			x	x		
Doç. Dr. Emin Murat CANGER	Ağız, Diş ve Çene Radyolojisi	E.Ü. Diş Hek.Fak.	x			x	x		
Doç. Dr. Mehmet DOLANBAY	Kadın Hast. ve Doğum	E.Ü. Tıp Fak..	x			x	x		
Doç. Dr. Fatih KARDAŞ	Çocuk Sağ. ve Hast.	E.Ü. Tıp Fak..	x			x	x		
Doç. Dr. Serpil TAHERİ	Tıbbi Biyoloji	E.Ü. Tıp Fak..		x		x	x		
Doç.Dr.Zafer SEZER	Farmakoloji	E.Ü. Tıp Fak.	x			x	x		
Doç. Dr. Adnan BAYRAM	Anest ve Rean.	E.Ü. Tıp Fak.	x			x	x		
Doç. Dr. Hakan İMAMOĞLU	Radyoloji	E.Ü. Tıp Fak.	x			x	x		
Dr.Öğr.Üyesi Oktay BOZKURT	İç Hastalıkları	E.Ü. Tıp Fak.	x			x	x		
Dr.Öğr.Üyesi Kemal Erdem BAŞARAN	Fizyoloji	E.Ü. Tıp Fak.	x			x		x	
Av. Tuğba TANRIVERDİ	Avukat	E.Ü. Tıp Fak.		x		x	x		
Ecz. Şükran TERZİ	Eczacı	Serbest Eczacı		x		x	x		
Sevtap KOÇER	Sivil Üye	Serbest		x		x	x		

*: Toplantıda Bulunma

Etik Kurul Başkanının
Ünvanı/Adı/Soyadı: Prof. Dr. Sema Kader KÖSE
İmza:

Not: Etik kurul başkanı, imzasının yer almadığı her sayfaya imza atmalıdır

KLİNİK ARAŞTIRMALAR ETİK KURULU KARAR FORMU (2011 - KAEK-80)

ARAŞTIRMANIN AÇIK ADI	Preoperatif Duygusal Stres Ölçeği (Kısa Form): Türkçe Geçerlik ve Güvenirlik Çalışması
VARSA ARAŞTIRMANIN PROTOKOL KODU	

ETİK KURUL BİLGİLERİ	ETİK KURULUN ADI	ERCİYES ÜNİVERSİTESİ KLİNİK ARAŞTIRMALAR ETİK KURULU
	AÇIK ADRES	Erciyes Üniversitesi Tıp Fakültesi Dekanlığı Melikgazi/KAYSERİ
	TELEFON	0 352 437 49 10 - 11
	FAKS	0 352 437 52 85
	E-POSTA	serifeserim@erciyes.edu.tr

BAŞVURU BİLGİLERİ	KOORDİNATÖR / SORUMLU ARAŞTIRMACI UNVANI / ADI / SOYADI	Doç. Dr. Özlem Ceyhan	
	KOORDİNATÖR SORUMLU ARAŞTIRMACININ UZMANLIK ALANI	İç Hastalıkları Hemşireliği	
	KOORDİNATÖR / SORUMLU ARAŞTIRMACININ BULUNDUĞU MERKEZ	Erciyes Üniversitesi, Sağlık Bilimleri Fakültesi, Kayseri	
	VARSA İDARİ SORUMLU ÜNVANI/ ADI SOYADI		
	DESTEKLEYİCİ		
	PROJE YÜRÜTÜCÜSÜ UNVANI/ADI/SOYADI (TÜBİTAK vb. gibi kaynaklardan destek alanlar için)		
	DESTEKLEYİCİNİN YASAL TEMCİLCİSİ		
	ARAŞTIRMANIN FAZI VE TÜRÜ	FAZ 1	
		FAZ 2	
		FAZ 3	
		FAZ 4	
		Gözlemsel ilaç çalışması	
		Tıbbi cihaz klinik araştırması	
		İn vitro tıbbi tanı cihazları ile yapılan performans değerlendirme çalışmaları	
		İlaç dışı klinik araştırma	
		Diğer ise belirtiniz	Bireysel Araştırma Projesi
	ARAŞTIRMAYA KATILAN MERKEZLER	TEKMERKEZ [x] ÇOKMERKEZ [ ]	ULUSAL [x] ULUSLARARASI [ ]

ASLI GİBİDİR

Şerife SERİM
Etik Kurul Sekreteri

Etik Kurul Başkanının
Ünvanı/Adı/Soyadı: Prof. Dr. Sema Kader Köse
İmza:

Not: Etik kurul başkanı, imzasının yer almadığı her sayfaya imza atmalıdır
